# Expression of synaptic proteins and development of dendritic spines in fetal and postnatal neocortex of the pig, the European wild boar *Sus scrofa*

**DOI:** 10.1007/s00429-025-02900-0

**Published:** 2025-02-07

**Authors:** Eric Sobierajski, Katrin Czubay, Marc-André R. Schmidt, Sebastian Wiedenski, Sarah Rettschlag, Christa Beemelmans, Christoph Beemelmans, Petra Wahle

**Affiliations:** 1 Faculty of Biology and Biotechnology, Developmental Neurobiology, Ruhr University Bochum, Bochum, 44870 Germany; 2 Regionalverband Ruhr Grün, Forsthof Üfter Mark, Forsthausweg 306, Schermbeck, 46514 Germany

**Keywords:** NMDA receptor, GABA receptor, PSD95, GRIP1, GAD-65/67, Fast-spiking interneurons, Porcine

## Abstract

Synapse formation is a critical step in neuronal development. Current knowledge is largely based on altricial rodents where synapse formation and maturation proceed largely postnatally. In precocially born mammals such as guinea pig presynapse and spine formation start well before birth. Here, we analysed the developmental expression of proteins associated with synapse formation and maturation together with the development of basal dendritic spines of pyramidal neurons of visual and somatosensory cortex of the pig, an emerging translational model for human neurodegenerative disorders. A total of 23 selected proteins was quantified with Western blots. Most were detectable from midgestation embryonal day (E) 65 onwards. About half reached the expression level seen at postnatal day (P) 90 pig already two weeks before birth (gestation 114 days) in somatosensory, albeit not yet in visual cortex. For instance, major molecular components of synaptic plasticity such as GluN2B, CamKIIα, α-actinin-2, synaptopodin and T286 phosphorylated CamKIIα were expressed at E100 in somatosensory cortex. Dendritic spine type quantification with DiI-labeled material revealed an increase of total dendritic protrusions from E70 onwards. The increase was steepest in somatosensory cortex which had, at E110, a proportion of mushroom spines equal to the proportion present at P90. Together, matching the ungulate life history, a rapid development of functional synaptic connectivity in prenatal somatosensory cortex serves the somatomotor abilities essentially required by the newborn nest-fledgling.

**Supplementary Information:**

The online version contains supplementary material available at 10.1007/s00429-025-02900-0.

## Introduction

The development of the pig cerebral cortex is mainly a prenatal process. Ungulates are precociously born with their senses and motor system functional because they need to run with their mother quickly after birth. As expected, myelination commences early during pregnancy in precocial animals like pig (Sobierajski et al. 2023), guinea pig (Tolcos et al. 2011), cattle (Urban et al. 1997), and sheep (Barlow 1969). When comparing temporal expression profiles, we find that myelin basic protein and myelin-associated glycoprotein are expressed around two weeks earlier in somatosensory cortex (SC; the representation of the rostrum has been analyzed) than in visual cortex (VC). Further, myelinated fibers are thicker in SC than in VC at the same age. Also, the axons of parvalbumin-expressing interneurons are more likely to be myelinated in SC than in VC at the same age (Sobierajski et al. 2023). This seems to be in line with the “cascading” hypothesis (Huttenlocher and Dabholkar 1997) of neurodevelopment: somatosensory representations develop early and somehow trigger the formation of functional maps serving the other senses. Based on receptor binding assays, the opposing “integrative network” hypothesis claims that cortical fields follow a concurrent developmental trajectory (Lidow et al. 1991), at least from birth onwards. Indeed, the blood vessel system of the pig cortex develops at the same time in SC and VC, and expression profiles of angiogenesis- and tight junction-related proteins reveal no temporal difference between SC and VC (Sobierajski et al. 2024). Angiogenesis in postnatal cortex has an activity-dependent component (Fonta and Imbert 2002; Tieman et al. 2004). Indeed, very early forms of spontaneous activity have been reported in cortex (Powell et al. 2024). It could suggest that such spontaneous and sensory-evoked activity might influence angiogenesis in fetal pig cortex. Overall, to our knowledge, time-dependent area differences e.g. of protein expression in early cortical development are understudied and literature evidence is scarce.

The two opposing findings (Sobierajski et al. 2023, 2024) raised the question of whether expression of developmentally regulated neuronal proteins might also display time-dependent area differences. If the SC matures more rapidly than the VC one would expect a higher level of proteins for neuronal function, e.g. for spines, presynapses, transmitter receptors, in the SC and a delayed expression in VC. Besides humans, knowledge about development and maturation of pre-and postsynaptic components has so far been obtained mainly from altricial rodents. Studies also describe dendritic spine morphology, density, and dynamics in diverse neuronal populations and cortical areas (reviewed by Moyer and Zuo 2018). For instance, spines from layer 5 pyramidal neurons in SC and auditory cortex of young mice are more dynamic than those in VC. Yet, the stability of mature mushroom spines and immature filopodia is comparable in the areas examined (Majewska et al. 2006). Further, in the same cortical area, layer 2/3 pyramidal cells have more spines along the apical dendrite than layer 5/6 pyramidal cells in both, motor and somatosensory cortex from P14 to P120 (Tjia et al. 2017). In pig, stellate neurons of layer 4 in SC and auditory cortex are more complex than those in VC at P3, and this reverses at 8 postnatal weeks (Jarvinen et al. 1998). Spine densities do not differ between the cortical areas at the two ages (Jarvinen et al. 1998).

Developmental studies in altricial species often focus on the critical period of sensory plasticity and malleability after birth. Precocial species such as guinea pig, ungulates and primates, and human with their perinatal brain development (possibly not as altricial as long believed; Gómez-Robles et al. 2024), have the period of rapid spine and synapse formation in the late fetal stage (Bourgeois 1997; Huttenlocher and Dabholkar 1997; Nacher et al. 2000). This explains why fetal growth restriction in these species substantially impairs cognitive abilities later in life. The fetal brain is constantly exposed to specific somatosensory input evoked by movements of the dam, of its siblings, or its own spontaneous muscle activity. In visual system of rodent and ferret, spontaneous retinal activity before eye opening drives the axonal wiring (Godement et al. 1984; Meister et al. 1991; Akerman et al. 2002; Adelsberger et al. 2005), and in primate and cat, eye-specific segregation is completed before birth (Rakic 1976; Sretavan and Shatz 1986). Functionally, in newborn sheep, the response properties of visual cortical cells such as orientation selectivity, receptive field orientation, and ocular dominance distribution are indistinguishable from those in the adult; just that binocular cells are initially equally well responding to monocular stimuli (Clarke et al. 1979) supporting the view that mature binocular interactions depend on visual experience. Having so little information on prenatal development and having the two opposing findings on temporal differences of development of myelin-related proteins but not of angiogenesis-related proteins (Sobierajski et al. 2023, 2024) prompted the present study on quantitative development of spines visualized with lipophilic dyes and synaptic proteins with Western blots in fetal and postnatal material of SC and VC of the pig.

## Materials and methods

### Animal material

The material is from our fetal pig brain collection (Ernst et al. 2018; Sobierajski et al. 2022). Our model of choice is a non-domesticated form, the European wild boar, *Sus scrofa* (Linné 1758) which has an approximately 41% larger brain weight compared to domestic landrace pig (Böndel 2017) without signs of domestication such as reduced sensory organs or deformed cerebellum. Fetuses derived from mostly young (first pregnancy) sows and piglets individually hunted for population control in accordance with the German Game Law (Jagdrecht) or killed in road accidents. The law requests disposal of viscera including sexual organs. During evisceration, the uteri were examined for pregnancies. As described in detail (Sobierajski et al. 2022), most fetuses were dissected at the Forsthof Üfter Mark, embryonic membranes were removed, and fetuses immersed in cold 4% paraformaldehyde (PFA) in 0.1 M phosphate buffer pH 7.4, or in cold Ringer solution for sampling raw material for blots. The P5 domestic German Landrace piglet was donated by Prof. Martin Schmelz and Prof. Dr. Maren Engelhardt, Institutes of Physiology and Anatomy, Medical Faculty, University Mannheim. Fine dissection of brain and body organs was done after transport to Ruhr University. Staging has been done using the crown-rump-length using the published formula for European wild boar (Henry 1968) and by external features.

**Table 1:** Antibodies and reagents for staining and protein blotting

Primary antibodies	Species, label, method	Source, order number, RRID	Dilution
α-Actinin-2	Rabbit, WB	Invitrogen, Carlsbad, Cat# PA5-27863 CA, USA, RRID: AB_254533	1:750
β-actin (housekeeping protein)	Mouse, WB	Sigma-Aldrich, St. Louis, MO, USA, Cat# A1978, RRID: AB_476692	1:6000
βIII-tubulin (housekeeping protein)	Mouse, WB	Sigma-Aldrich, St. Louis, MO, USA, Cat# T8660, RRID: AB_477590	1:3000
Caspase-3	Rabbit, WB	Cell Signaling, Cambridge, UK, Cat# 9662, RRID: AB_331439	1:1000
CaMKIIα	Mouse, WB	Abcam, Cambridge, UK, Cat# ab22609, RRID: AB_447192	1:1000
pCaMKIIα T286	Mouse, WB	Invitrogen, Carlsbad, CA, USA, Cat# MA1-047, RRID: AB_325402	1:1000
GABA_A_Rα1	Mouse, WB	NeuroMab, Davis, CA, USA, Cat# 75–136, RRID: AB_ 2,877,288	1:1000
GABA_A_Rβ3	Mouse, WB	NeuroMab, Davis, CA, USA, Cat# 75–149, RRID: AB_ 2,109,585	1:500
GAD-65/67	Mouse, WB	Enzo Life Sciences Inc., Farmingdale, NY, USA, Cat# ADI-MSA-225, RRID: AB_ 2,039,129	1:1000
GluA2	Mouse, WB	NeuroMab, Davis, CA, USA, Cat# 75 − 002, RRID: AB_ 2,232,661	1:500
GRIP1	Rabbit, WB	Synaptic Systems, Göttingen, DE, Cat# 151,003, RRID: AB_10804287	1:1000
Homer-1	Rabbit, WB	Synaptic Systems, Göttingen, DE, Cat# 160,033, RRID: AB_887730	1:1000
Kv3.2	Rabbit, WB	Alomone Labs Ltd, Jerusalem, IL, Cat# APC-011, RRID: AB_20401686	1:1000
Munc-18	Rabbit, WB	Synaptic Systems, Göttingen, DE, Cat# 116,003, RRID: AB_2619783	1:1000
NMDAR subunit 1 (NR1/GluN1)	Mouse, WB	NeuroMab, Davis, CA, USA, Cat# 75–327, RRID: AB_2315839	1:500
NMDAR subunit 2B (NR2B/GluN2B)	Rabbit, WB	Millipore, Burlington, MA, USA, Cat# 06-600, RRID: AB_310193	1:1000
PSD95	Mouse, WB	Synaptic Systems, Göttingen, DE, Cat# 124,011, RRID: AB_10804286	1:1500
p38/ Synapophysin	Mouse, WB	Synaptic Systems, Göttingen, DE, Cat# 101,011, RRID: AB_887824	1:5000
p70/Synapsin1	Mouse, WB	Synaptic Systems, Göttingen, DE, Cat# 106,001, RRID: AB_887805	1:2000
RIM1	Rabbit, WB	Synaptic Systems, Göttingen, DE, Cat# 140,003, RRID: AB_AB_887774	1:1000
Synaptopodin	Rabbit, WB	Synaptic Systems, Göttingen, DE, Cat# 163,002, RRID: AB_887825	1:1000
Synaptotagmin-2	Rabbit, WB	Synaptic Systems, Göttingen, DE, Cat# 105,123, RRID: AB_2199465	1:1000
TOM70	Mouse, WB	Santa Cruz Technologies, Cat# Sc-3,900,545, RRID: AB_2714192	1:1000
Trim46	Rabbit, WB	Synaptic Systems, Göttingen, DE, Cat# 377,008, RRID: AB_2905572	1:2500
**Secondary antibodies**			
Anti-mouse	Rabbit, alkaline phosphatase, WB	Dako A/S, Glostrup, DEN, Cat# D0314	1:5000
Anti-rabbit	Goat, alkaline phosphatase, WB	Dako A/S, Glostrup, DEN, Cat# D0487, RRID: AB_2617144	1:3000
**Reagents**			
DiIC18(3)	Fluorescent lipophilic dye	Molecular Probes via Invitrogen, Carlsbad, CA, USA,	crystalline
DAPI	Fluorescent counterstain	Sigma-Aldrich, St.Louis, MO, USA, Cat# D9542	1:3000

### Tissue processing and DiI staining

As described (Ernst et al. 2018; Sobierajski et al. 2022) dissected brains were immersion-fixed in 4% paraformaldehyde in 0.1 M phosphate buffer (PB) pH 7.4 with 5% (vol/vol) water-saturated picric acid for about 2 weeks at 8 °C (refreshed once). For DiI staining, small slabs of tissue containing one or two gyri were razor-cut from the VC and SC and freed from remaining meninges. Using glass micropipettes, small crystals of DiI were implanted into gray matter (layers 3–5 at E110 and P90) to label nearby pyramidal cells via their horizontal axon collaterals. Tissue slabs were stored in fixative in the dark at 4 °C for at least two weeks to allow dye diffusion. Slabs were cut with a vibratome (MA752, Campden Instruments) into 80–120 μm thick sections. The E70, E85, and E90 tissue blocks were pre-embedded in 1% agarose to improve stability during slicing. The sections were stained with DAPI for 10 min, washed repeatedly, mounted on silanized slides, and coverslipped with ImmuMount.

### Protein blots

Tissue blocks containing gray and upper white matter from VC and SC were taken during preparation of the brains (ages E65, E80, E95, E100, P90) as described (Sobierajski et al. 2023). VC and SC of P60 rat cortex were used as positive control. Tissue blocks were pulverized on dry ice. A microspoon-sized powder sample was homogenized in standard RIPA lysis buffer. The amount of protein was determined with the Markwell assay. Proteins were separated on 8%, 10%, or 14% polyacrylamide gels (30 µg protein per lane) at 7 mA per gel overnight. Proteins were transferred to nitrocellulose membranes (BA 85/Protran, 0.2 μm pore size, Schleicher and Schuell or Amersham) with a tank blotter (Hoefer, San Francisco, CA, USA). Smaller proteins were transferred with buffer containing 20% MeOH and no SDS, proteins above 70 kDa were transferred with buffer containing 15% MeOH and 0.05% SDS. The blots were stained with Ponceau-Red to visualize major protein bands. Membranes were then separated into horizontal stripes containing the desired proteins (up to 10 per gel; Engelhardt et al. 2018) and blocked with TBS (50 mM Tris, pH 7.4, 150 mM NaCl) containing 5% bovine serum albumin for 1 h. Primary antibodies (Table 1) were incubated overnight at 4 °C, followed by washes for 10 min each in TBS, TBST (50 mM Tris, pH 7.4, 150 mM NaCl, 0.1% Tween-20), and TBS. Matching alkaline phosphatase-conjugated secondaries (Table 1) were incubated for 90–120 min. Blots were stained with 0.18 mg/mL BCIP and 0.35 mg/mL NBT, washed with water, and dried.

Several other antibodies have been tried. While all were staining the target band in rat cortex, some did not deliver reliably measurable bands in pig. Nevertheless, we listed these antibodies in Online Resource 2 to inform future research.

### Analysis

DiI-stained sections were imaged with a Leica TSC SP5 confocal microscope (10x, 40x, and 63x objective with 1.1 NA, 1024 × 1024 px). Raw confocal images were deconvolved with Huygens Essential (Scientific Volume Imaging B.V.) to improve image stack quality and remove background noise. Selected dendrites were optically isolated with the surface tool and a masking algorithm of Imaris 10.1. software (Oxford Instruments). The “filament” algorithm in Imaris allows 3D reconstruction. The initial semi-automatic reconstruction was strictly followed by manual work-over and classification of spine types in selected categories following established criteria (Risher et al. 2014). This often involved two experienced observers equipped with the only reliable high-tech, the human retina and the human brain, to make concise decisions regarding the continuum of spine shapes and their intermediates known to exist during development and even in adulthood (Ofer et al. 2022). Spines along secondary and tertiary basal dendrites of pyramidal cells of infragranular layers were reconstructed. For documentation, global whole-picture contrast-, brightness-, color intensity- and saturation settings were adjusted with Adobe Photoshop^®^. Scale bars were generated with Leica Acquisition Suite X and inserted with Adobe Photoshop^®^ (CS6 Extended, Version 13.0 × 64). Data management was done in Microsoft Excel 365. Graphs were made with SigmaPlot 12.3 (Systat Software GmbH). Plots and images were arranged with Adobe Photoshop^®^. Blots were photographed and relative band intensities were determined with ImageJ. All protein bands were normalized to the 42 kDa β-actin or the 55 kDa βIII-tubulin which served as control for loading equivalency. Values were then expressed relative to the intensity at P90. P90 is shortly before puberty onset in pig and has been taken as equivalent to early adolescence (Sobierajski et al. 2022), although development still continues towards full adulthood which is reached at about two years (Brogi et al. 2021). Statistical analysis of the spine density was done with a non-parametric Mann-Whitney rank sum test.

## Results

### Developmental expression of neural and synaptic proteins

We selected proteins which, in rodent, are developmentally regulated and/or related to neuronal plasticity. Blot by blot we aimed to compare expression profiles in VC to SC beginning at midgestation E65, just after the begin of gyrification (Ernst et al. 2018), at late gestation E80, E95, E100, and at P90. Levels at P90 had been set to 1.

**Fig. 1:**
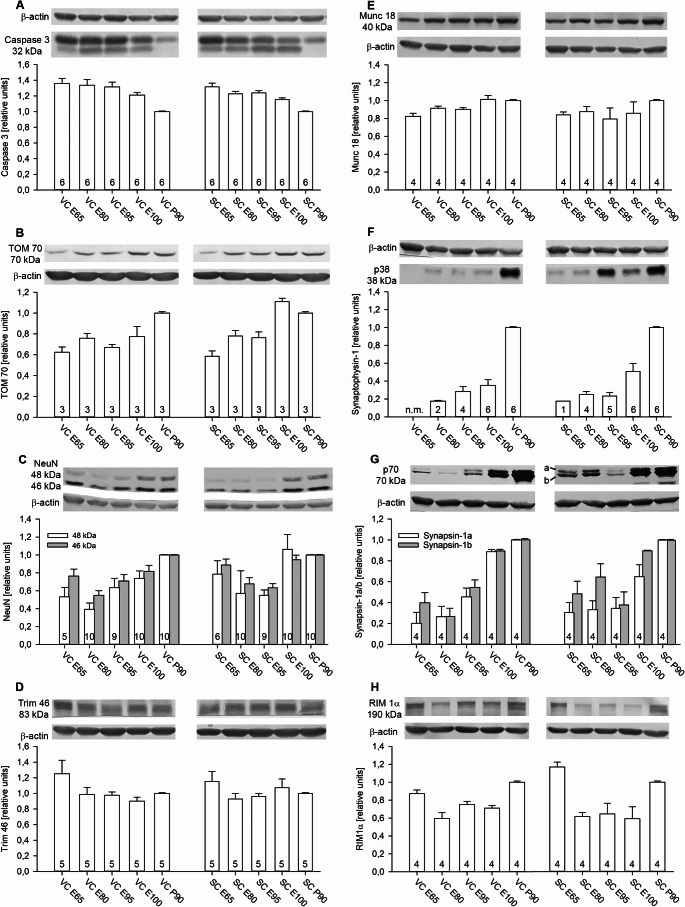
Developmental expression of neural and synaptic proteins. (**A**) Cell-death-related caspase-3. (**B**) Mitochondrial marker translocator of outer membrane, TOM70. (**C**) Neuron-specific RNA splice factors, NeuN. (**D**) Neuronal polarity and axon initial segment marker Trim46. (**E**) Presynaptic organizer Munc-18. (**F**) Presynaptic vesicle protein p38/synaptophysin. (**G**) Presynaptic vesicle protein p70/synapsin-1a/b. (**H**) Presynaptic organizer RIM1. β-actin at 42 kDa was used for normalization

Caspase-3 expression indicates cell death which is high early in development. As expected, expression was high early on in VC declining after E95. Decline in SC commenced already after E65. Lowest level was seen at P90 (Fig. 1A). Translocase of the outer membrane, TOM70, works in a complex to transport proteins into mitochondria. It is a general metabolic marker useful for assessing the biogenesis of mitochondria (Kreimendahl and Rassow 2020; Ma et al. 2023). Given the dramatic volume increase of the cortex from midgestation to postnatal and the rapid differentiation of energy-hungry neurons as well as the addition of microglia, myelin-forming oligodendrocytes, and blood vessels (Ernst et al. 2018; Sobierajski et al. 2022, 2023, 2024) we expected an increase. Indeed, TOM70 expression increased substantially reaching the postnatal P90 level at E100 in SC, albeit not yet in VC (Fig. 1B). Looking more at neurons, the neuron-specific RNA splice factor NeuN comes in two isoforms of 46 and 48 kDa. The larger isoform was faintly expressed at E65 (Fig. 1C). Both isoforms increased from E80 onwards, and the P90 level was reached at E100 in SC, albeit not yet in VC. The E65 levels were higher than levels at E80 and E95 resulting, unexpectedly, in a U-shaped expression profile (Fig. 1C). The microtubule-associated Ring finger E3 ligase protein Trim46 is involved in neuronal polarity and axonal outgrowth (Curcio and Bradke 2015). Expression was highest at E65 remaining at a constant level thereafter (Fig. 1D). The enrichment of Trim46 in the axon initial segment maintains neuronal polarity and it is in line with the presence of βIV-spectrin-positive axon initial segments in pig cortex as early as E70 (Ernst et al. 2018).

Growing axons secrete transmitters, and since synaptic vesicle proteins become selectively delivered to axons (Watson et al. 2023) their expression has been taken as a proxy for development of axonal connectivity. The vesicle docking and fusion protein Munc-18 has a crucial role in exocytosis. Already from E65 onwards VC and SC levels were comparable to those at P90 (Fig. 1E). Munc-18 is involved in organizing the presynapse rather than being a marker for synaptogenesis since it was already highly expressed when the vesicle-associated proteins p38 and p70 were still very low. Yet, transmitter release from nascent axons can occur without molecularly mature vesicles (Andreae and Burrone 2014). P38/synaptophysin is a marker for presynapse development. It was present weakly in SC at E65 but not yet in VC at E65. Protein amounts increased slowly until E100 and then doubled to P90 (Fig. 1F). Similarly, p70/synapsin-1a/b was detectable at E65 with higher levels in SC than in VC and increasing in both areas (Fig. 1G). Another presynaptic active zone protein is RIM1. The expression profile was U-shaped in VC and SC. Levels at E100 were not yet near P90 levels in both areas (Fig. 1H).

**Fig. 2:**
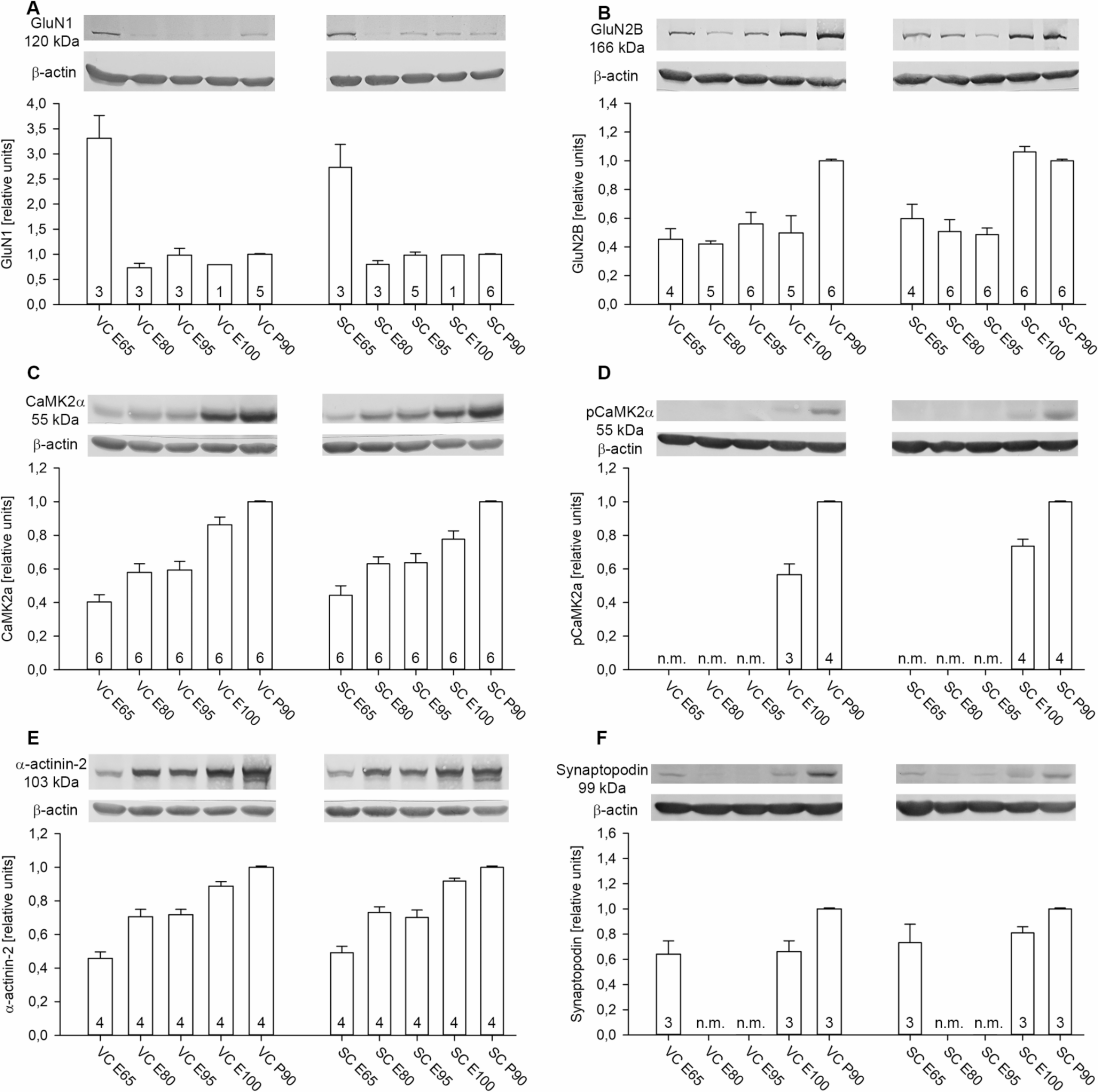
Developmental expression of plasticity-related proteins. (**A**) NMDAR subunit GluN1. (**B**) NMDAR subunit GluN2B. (**C**) Postsynaptic enzyme CamKIIα, total protein. (**D**) T286 phosphorylated CamKIIα. (**E**) Structural plasticity-related actin bundling protein α-actinin-2. (**F**) Spine apparatus and cisternal organelle-associated synaptopodin. β-actin at 42 kDa was used for normalization. n.m., the protein was not measurable at this age in this cortex

**Fig. 3:**
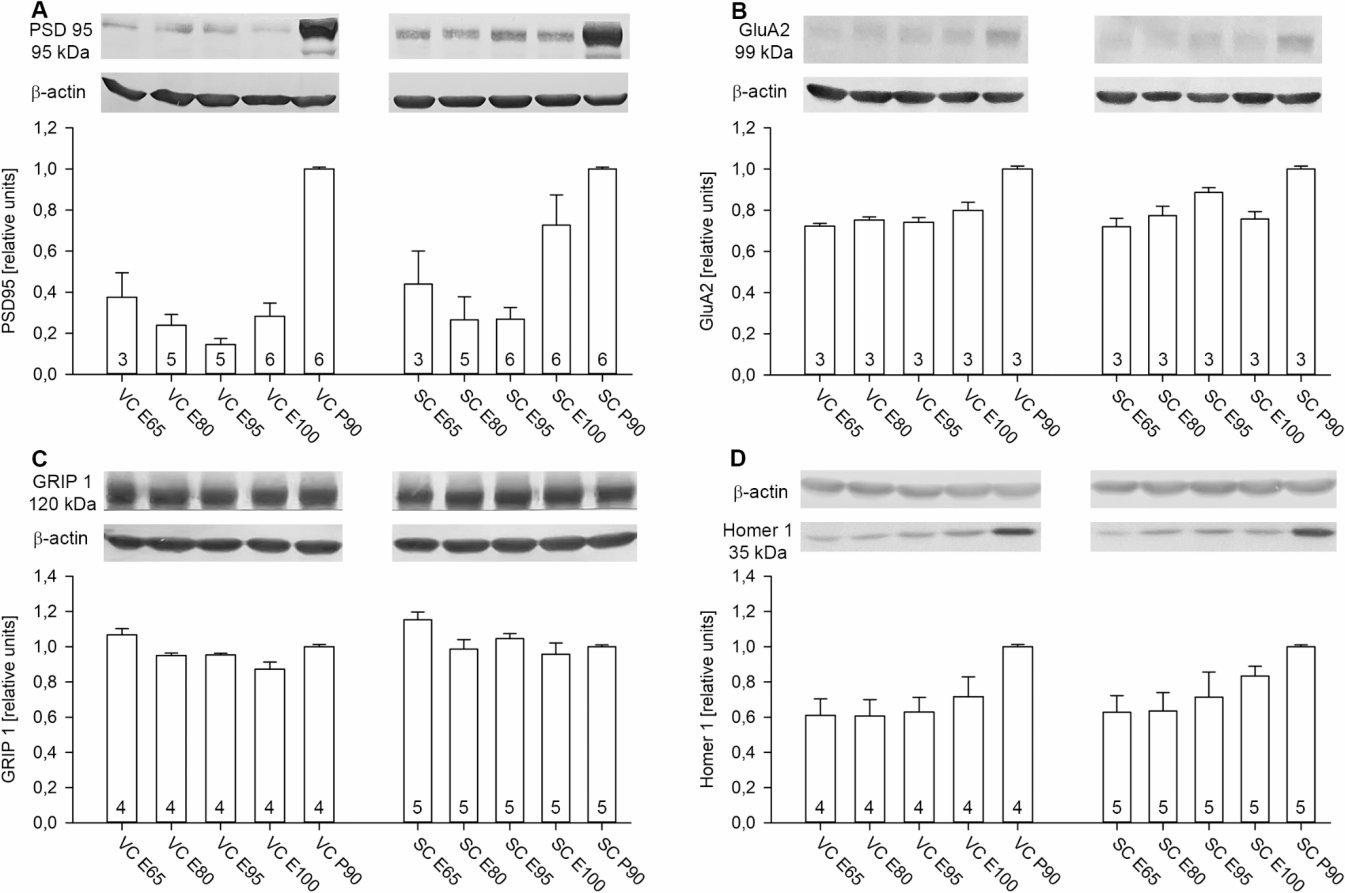
Developmental expression of proteins of the excitatory synapse. (**A**) Organizer of the excitatory postsynapse, postsynaptic density protein PSD95. (**B**) AMPA-type glutamate receptor GluA2. (**C**) AMPA receptor interacting protein GRIP1. (**D**) Protein crosslinking metabotropic glutamate receptors to intracellular mediators, Homer-1. β-actin at 42 kDa was used for normalization

**Fig. 4:**
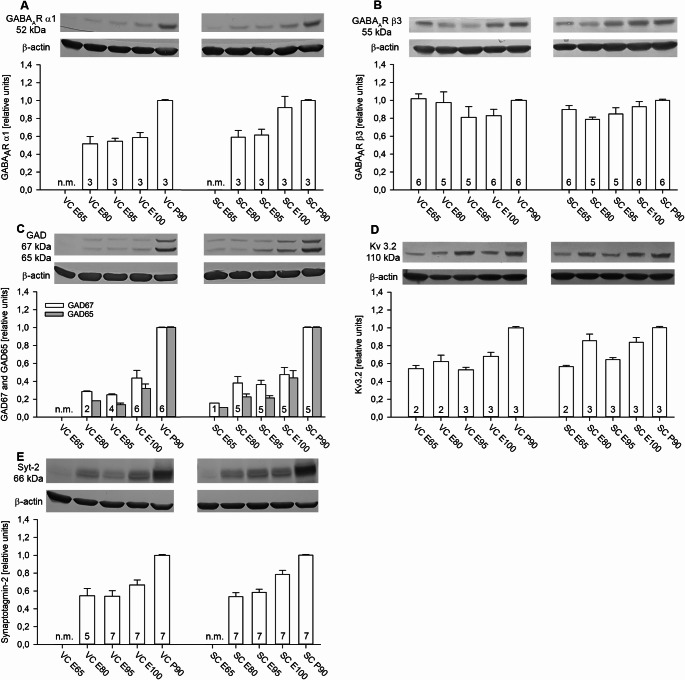
Developmental expression of proteins of the inhibitory synapse. (**A**) GABA_A_Rα1. (**B**) GABA_A_Rβ3. (**C**) Glutamate decarboxylase isoforms 65 and 67 kDa, GAD-65/67. (**D**) Voltage-gated potassium channel Kv3.2. (**E**) Calcium sensor in axon terminals of fast-spiking interneurons, synaptotagmin-2. β-actin at 42 kDa was used for normalization. n.m., the protein was not measurable at this age in this cortex

We also compared P90 pig VC to rat VC (Online Resource 1 A-D) by using blot images not included in Figs. 1, 2, 3 and 4. The aim was to confirm the quantification with independent blots and to explicitly focus on the delayed development of the VC. The comparison revealed that NeuN, Trim46, Munc-18, p70/synapsin-1, and RIM1 expression were fairly similar to rat in terms of postnatal band intensity and molecular weight. Pig caspase-3 ran lower than rat caspase-3 and the smaller isoform seen in pig up to E100 has not been reported in rodent. TOM70 had a doublet band in rat lysates but not in pig lysates. Rat p38/synaptophysin ran slightly lower than pig p38 (Online Resource 1 A).

In Figs. 1, 2, 3, and 4, VC is left, SC is right, each with 5 stages from embryonic day (E) 65, E80, E95, E100 to postnatal day P90 the average of which has been set to 1. Numbers in the bars indicate the number of independent lysates that delivered measurable bands. n.m., not measurable; the protein of interest was not detectable in these lysates. In case a lysate was run twice for a given marker, the average has been included to avoid technical repeats. Bars represent the mean with S.E.M.. Representative gel stripes are arranged together with the housekeeping protein from the same lanes. Molecular weight [kDa] is given.

Before describing the other proteins, we shall briefly address the expression profiles. Seeing more intense bands with age, as with TOM70, p38/synaptophysin, and p70/synapsin-1a/b, is commonly interpreted as an increase of protein amounts. This has been also the case for the expression of myelin-related proteins and GFAP detected with blots from the very same material (Sobierajski et al. 2023). Seeing less intense bands, as with caspase-3 or Trim46, is commonly interpreted as a decrease. This has been also the case with PDGFRα, a marker for immature oligodendrocytes (Sobierajski et al. 2023). In contrast, U-shaped profiles as revealed for NeuN, RIM1, or PSD95 (Fig. 3) look peculiar. It might suggest a transient expression, or transient interaction with binding partners masking the antigenic epitope, up to harvest at a wrong circadian time point or simply postmortem protein degradation. Myelin proteins are particularly susceptible to the latter, however, within our postmortem periods (Ernst et al. 2018) we could not detect any degradation (Sobierajski et al. 2023). Moreover, such interpretations make little sense in case of NeuN and PSD95 which are continuously increasing in neurons with ongoing differentiation without regulation by circadian mechanisms. Rather, we have to consider the dramatic volume increase of the brain from ~ 5 g at E45 to ~ 90 g at P90 (Ernst et al. 2018; Sobierajski et al. 2023). At E65, the future cortical gray matter consists mostly of densely packed immature neurons and the still-migrating neurons of the cortical plate. Microglia (Sobierajski et al. 2022) and immature oligodendrocytes are still scarce, and astrocytic GFAP protein expression is barely detectable (Sobierajski et al. 2023). Therefore, at E65, neuronal proteins expressed by millions of neurons will be the dominant proteins in a lysate of 30 µg total protein. With time, these early expressed neuronal proteins become diluted because proliferating, invading and/or maturating microglia, macroglia, and endothelial cells contribute more and more non-neuronal proteins to the lysate. Accordingly, the band intensity drops transiently only to recover towards the postnatal stage with ongoing neuronal differentiation.

We therefore suggest an alternative interpretation. U-shaped profiles are seen with proteins that are expressed at substantial amounts early on and which then remain rather on a plateau. A constant protein level over the ages should be rather regarded as a continuous increase of expression with the “increase” being compensated for by the “dilution”, be it via neurons or glia which for instance also express many transmitter receptors. An increasing intensity of a band should be regarded as an even steeper increase of protein expression, again, in neurons or glial cells. The caspase-3 expression then would initially increase before declining. Reports on U-shaped expression profiles are rare presumably because midgestation brain samples are rarely analyzed. For instance, expression of protein tyrosine phosphatase 1D in fetal superior colliculus displays a U-shaped profile which has been interpreted as reflecting a switch in function (Reinhard et al. 2009). Increasing, decreasing and U-shaped profiles have also been shown for claudin-3, Jam-A, and claudin-12, respectively, in fetal sheep from 0.60 gestational age onwards (Sadowska et al. 2015).

### Developmental expression of plasticity-related proteins

The mRNA encoding the obligatory NMDA receptor subunit GluN1 increases in rat cortex from birth to peak levels during the fourth postnatal week followed by a decline towards adulthood (Hofer et al. 1994) and the protein increases about 3-fold from birth to P40 (Luo et al. 1996). In pig, however, the strongest expression was at E65 in VC and less so also in SC with levels being 3-fold higher than at P90 (Fig. 2A) confirming the view that this subunit is not limiting the assembly of NMDA receptors. At the following fetal ages, the GluN1 band became extremely faint and was not always measurable by densitometry in all lysates tested. The band was again well detectable at P90. With all caution towards measuring such a faint expression, the bar graphs did suggest a small increase from E80 to E95. In contrast, the synaptic plasticity-related subunit GluN2B was well detectable from E65 onwards in VC and SC. A steep increase to the postnatal P90 levels has occurred at E100 in SC, albeit not yet in VC (Fig. 2B). Of note, the expression of glutamate and GABA receptors may not solely mirror neuronal maturation since AMPA, NMDA and GABA_A_R are also expressed in macroglia (Gundersen et al. 2015; for review).

The 55 kDa CamKIIα subunit is expressed in pyramidal cells and upregulates in rodent VC around eye opening at the end of the second postnatal week. CamKIIα autophosphorylation is essential for NMDAR-dependent plasticity (Gordon et al. 1996; Glazewski et al. 2000). Interestingly, CamKIIα protein was expressed and steadily increased in pig VC and SC from E65 onwards (Fig. 2C) suggesting that CamKIIα mirrored the functional differentiation of excitatory neurons. Also important, the T286-phosphorylated form was detectable already at E100, a bit stronger already in SC than in VC. A substantially higher CamKIIα level was seen at P90 (Fig. 2D) suggesting a massive increase after E100. The CamKIIα interaction with α-actinin-2 is required for structural long-term potentiation (Curtis et al. 2023) and spine growth and stability. Indeed, α-actinin-2 was well expressed in pig VC and SC steadily increasing from E65 onwards (Fig. 2E). Also essential for spine maturation and maintenance is the spine apparatus with synaptopodin via its interaction with α-actinin-2. Synaptopodin comes as a long and a short transcript and PCR detects both in brain and in kidney (Asanuma et al. 2005). At the protein level, only the smaller, ~ 100 kDa isoform is expressed in brain. In pig, synaptopodin expression was U-shaped, being present at E65, not measurable at E85 and E95 and again present at E100 in SC almost comparable to P90. Synaptopodin expression levels at E100 in VC were less than at E100 in SC (Fig. 2F). Comparing pig VC to rat VC revealed the bands of GluN1, GluN2B, CamKIIα, α-actinin-2, and synaptopodin at the same molecular weight as in rat VC. The intensity of the P90 GluN1 and synaptopodin bands was lower in pig VC than in rat VC, which might reflect a difference in antibody binding (Online Resource 1B).

### Developmental expression of proteins of the excitatory synapse

The postsynaptic scaffold PSD95 anchors NMDA receptors and interacts with AMPA receptors. PSD95 displayed a mild U-shaped expression profile with a strong increase in SC at E100 which was not yet seen in VC (Fig. 3A), and higher expression at P90. It resembles the human where PSD95 is strongly expressed in the neonate increasing two-fold towards adolescence (Webster et al. 2011). We tested two GluA2-specific antibodies, both yielding only faint bands in pig VC and SC. With all caution, GluA2 was present at the fetal stages, and at higher levels at P90 (Fig. 3B). The glutamate receptor interacting protein GRIP1 is expressed postnatally in rodent cortex and parallels that of AMPA receptors. GRIP1 binds in particular GluA2 (Dong et al. 1999) and has an essential role in trafficking AMPA receptors into the postsynapse when cells undergo long-term potentiation. GRIP1 was strongly expressed from E65 onwards to postnatal P90 levels in VC and SC in line with the presence of GluA2 (Fig. 3C). Homer-1 is a postsynaptic scaffold protein, interacts with Shank proteins and links metabotropic glutamate receptors with intracellular signaling partners such as the IP3 receptor. Expression was present from E65 onwards, and steeply increasing between E100 to P90 in VC. Expression in SC was almost at postnatal P90 levels by E100 (Fig. 3D). Comparing pig to rat VC revealed PSD95, GluA2, and GRIP1 at the same molecular weight as in rat VC. GluN2A was at a lower intensity in pig VC than in rat VC, possibly reflecting a difference in antibody binding. Homer-1 ran slightly lower in pig than in rat VC (Online Resource 1 C).

### Developmental expression of proteins of the inhibitory synapse

GABA_A_Rα1 and β3 are major components of GABA receptors on pyramidal neurons. The mRNAs encoding the two subunits have been also reported in oligodendrocyte precursors in fetal and postnatal human brain (Gutierrez et al. 2023). GABA_A_Rα1 was not yet detectable at E65 in VC and SC but increased thereafter reaching P90 levels in SC earlier than in VC (Fig. 4A). This confirms protein blot data in pig parietal cortex at E100 to P7 with immunoreactivity being perinatally in pyramidal and nonpyramidal neurons (Kalanjati et al. 2017). GABA_A_Rβ3 was at the P90 level from E65 onwards in SC and very mildly U-shaped in VC (Fig. 4B). GAD-65/67 enzymes are expressed in inhibitory interneurons and synthesize GABA. Expression was not yet measurable at E65 in VC. In SC, the isoforms were detectable from E65 onwards and fetal expression levels were overall higher than in VC. Yet, the major increase towards the P90 level seemed to occur after E100 in both areas (Fig. 4C). Interneurons, in particular the fast-spiking types, express Kv3 channels for a fast repolarization of the action potential. Further, synaptotagmin-2 (Syt-2) is the presynaptic calcium sensor specifically in fast-spiking basket and chandelier cells. Kv3.2 was expressed from E65 onwards increasing steeper in SC than in VC (Fig. 4D). Syt-2 was not measurable at E65, but well present from E85 onwards. As with GAD-65/67, the major increase towards the P90 level occurred after E100 in both areas (Fig. 4E). Comparing pig to rat VC revealed the bands of GAD-65/67, Syt-2 and GABA_A_ receptors were at the same molecular weight and intensity as in rat VC. Kv3.2 expression levels tended to be higher in pig at P90. In terms of molecular weight, pig Syt-2 was running lower than rat Syt-2 (Online Resource 1D).

### Development of pyramidal cell dendritic spines and spine types

**Fig. 5:**
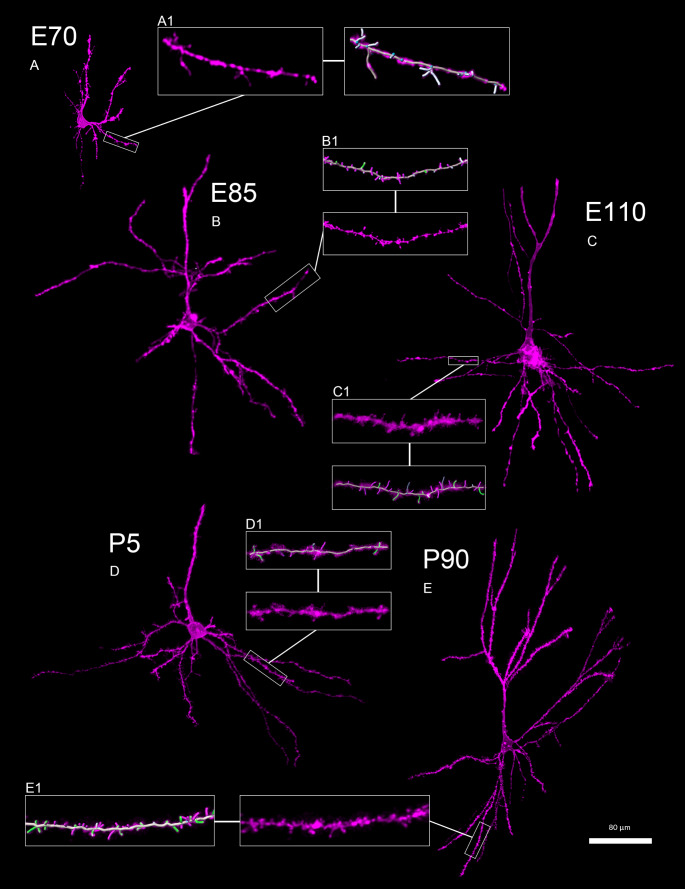
Development of pyramidal neurons in visual cortex from E70 to P90. **A-E**. Confocally imaged DiI-labeled pyramidal cells from VC. (**A**) Pyramidal neuron at E70. (**B**) Pyramidal neuron at E85. (**C**) Pyramidal neuron at E110. (**D**) Pyramidal neuron at P5 (domestic pig). (**E**) Pyramidal neuron at P90. A1-E1. A basal dendritic segment in higher magnification either without or with reconstructed spines; thin spines, magenta; stubby, light gray; mushroom, green; filopodia, dark gray. Scale bar is 80 μm

The density and morphology of dendritic spines are important indicators of the development of neuronal connectivity. Here, we acquired confocal scans of DiI-stained neurons from the VC and SC from E70 to P90 and reconstructed the basal dendrites, and classified the dendritic protrusions (addressed as ‘spines’ below). Only second and higher-order dendritic segments were included. Dendritic end segments were excluded as the thin distal tips of dendrites often have fewer spines. Thick proximal dendritic shafts were excluded because short spines may be masked by the thick shaft (Lendvai et al. 2000; Xu et al. 2020). At E70, neurons had small somata (Fig. 5A). They formed spines with immature morphologies, thus we decided to not determine spine types at this stage (Fig. 5A1). At E85, neurons had a much more mature morphology (Fig. 5B). Now, spine types were determined (Fig. 5B1). An increase in spine density proceeded between E85 and E110, and pyramidal cells developed larger somata with longer and more branched dendrites (Fig. 5C). Pyramidal neurons of the P5 domestic piglet did not obviously differ from E110 boar cells in morphology (Fig. 5D) but had a lower spine density (Fig. 5D1). Interestingly, volume, branching, and segment length of blood vessels of the P5 domestic piglet are somewhat lower than values obtained in E100/E110 wild boar marginal zone and gray matter. This suggests that vascular development also tends to proceed slower in domestic pig than in wild boar (Sobierajski et al. 2024). Until P90, the complexity of pyramidal neurons (Fig. 5E) had increased further, and the density of spines seemed to be subtly higher (Fig. 5E1).

**Fig. 6:**
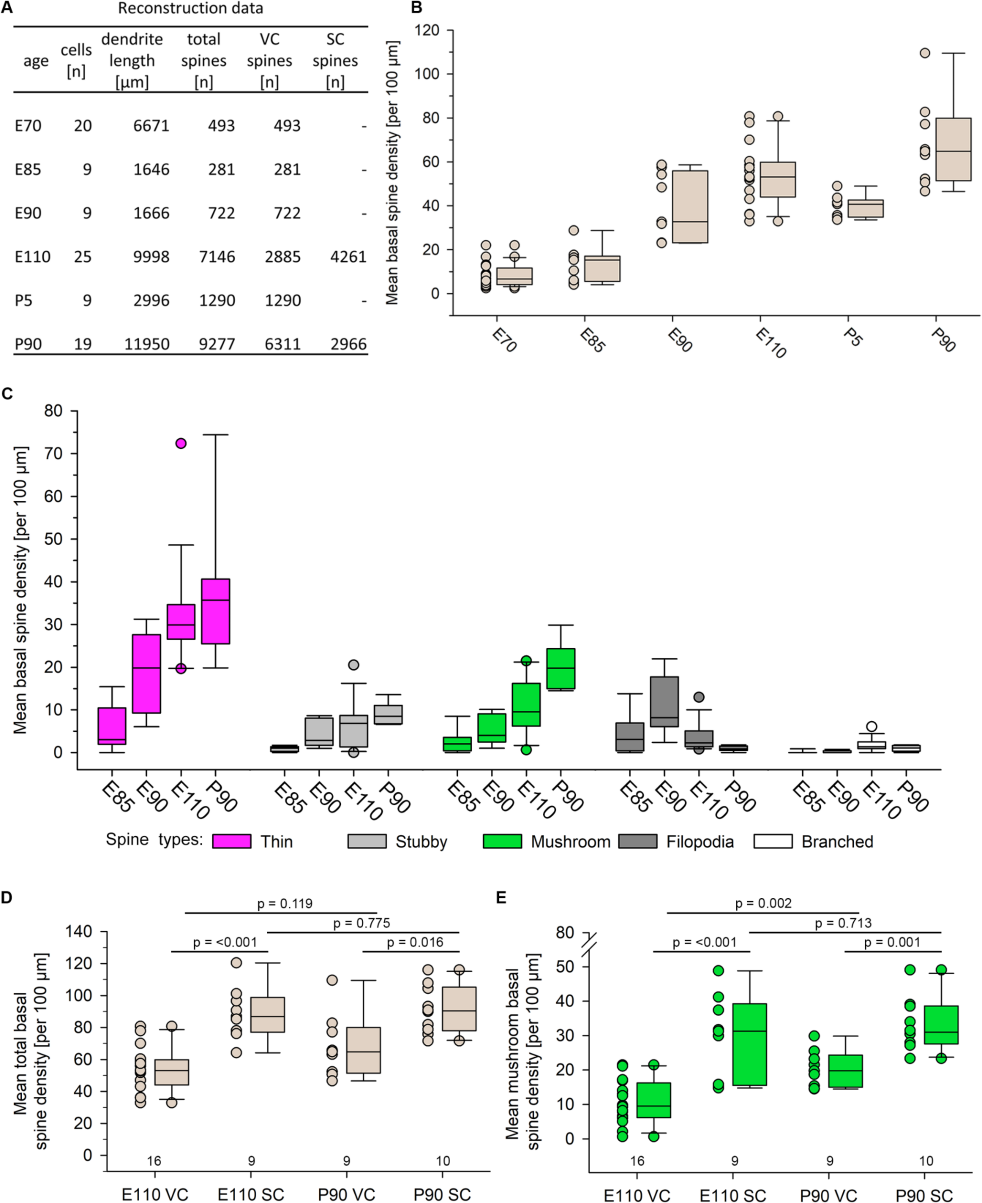
Assessment of spine density and composition of spine types. (**A**) Table summarizing the reconstruction data. (**B**) Total spine density from E70 to P90 in VC. (**C**) Spine types at E85, E90, E110 and P90 in VC; spine types are color-coded. (**D**) Interareal comparison of the total spine density at E100 and P90 in VC and SC. (**E**) Mushroom spine density at E100 and P90 in VC and SC. Note in D and E that total spine density and mushroom spine density in SC at E110 were at the level seen in SC at P90. For D and E, the n of cells are given below the boxes; *p* values have been determined with the Mann-Whitney rank sum test

Quantification of dendritic spines is shown in Fig. 6. Raw data are summarized in Fig. 6A. Differences in the sampling size between the respective stages have been due to the limited availability of the fetal brain tissue. Online Resource 3 contains more detailed information about the cells and dendrites in this analysis. In VC, from E70 to E85, total spine density increased on average from 8 to 18 spines per 100 μm (Fig. 6B). This was followed by a steeper increase to 39 spines per 100 μm at E90, and 53 spines per 100 μm at E110. As expected from the qualitative data, the P5 domestic piglet had a total spine density of 43 spines per 100 μm on average which was in the lower range of what was present in boar at E110. At P90, total spine density was on average 65 spines per 100 μm (Fig. 6B). At E85, the majority of spines were classified as thin and filopodial spines, mushroom and stubby spines were rare (Fig. 6C). Thin spines continued to be the dominant fraction at E90 and E110 and numbers were highly variable from cell to cell. The density of mushroom and stubby spines had increased. Filopodia increased to a maximum at E90 and declined towards E110 and P90. At P90, stubby spines remained at about the level seen at E110. Mushroom spines doubled with an increase from 9 spines per 100 μm at E110 to 19 spines per 100 μm on average at P90 (Fig. 6C).

In Fig. 6B, the almost three-fold variability of total spines observed at E110 and P90 might indicate large cell-individual differences in spine densities. This is known to occur in vivo in rat and ferret VC (Larkman 1991; Wahle et al. 2022). The blot data suggested area differences, which prompted us to examine the morphological correlates. At E110 and at P90, SC neurons had significantly higher total spine densities than VC neurons (Fig. 6D). In VC, P90 displayed a higher mean total spine density on average than E110, however the increase was not significant (Fig. 6D). In SC, the total spine density at E110 was already similar to the density at P90 (Fig. 6D). The advanced development of SC pyramidal neurons and the slower development of VC pyramidal cells, respectively, was clearly mirrored by the mushroom spine development. These are the most stable spines (Majewska et al. 2006), yielding quite compact boxes at E110 and P90 (Fig. 6C) without large whiskers and outliers. Thus, mushroom spines were suitable for an interareal comparison (Fig. 6E). In SC, the proportion of mushroom spines seen at P90 was reached already at E110. In VC, the proportion of mushroom spines was still low at E110 and increased significantly to P90. Overall, at both stages, mushroom spine densities in VC pyramidal neurons were lower than those in SC pyramidal neurons. This could suggest that spine densities of VC pyramidal neurons develop with a delay compared to SC pyramidal neurons. However, it could also suggest that VC pyramidal cells will always have lower spine densities than SC pyramidal cells given the behavioral importance of the pig’s somatosensory perception via the trunk compared to vision in a species most active at dawn and during the night. To this end, P90 marks the endpoint of our analysis, and it cannot be excluded that further changes in spine density or proportions occur in older individuals.

## Discussion

Our study delivered three major results. First, of the 23 activity-dependent and plasticity-related proteins tested here 18 were expressed from midgestation onwards. Second, they reached the P90 postnatal levels already at E100 in SC or displayed a steeper increase in SC than in VC. Third, basal dendrites of E110 SC pyramidal cells displayed total spine and mushroom spine densities similar to densities seen at P90, while E110 VC pyramidal neurons had not reached the densities seen at P90. The expression of several tested proteins (e.g. Synaptophysin-1, GluN2B, PSD95, GABA_A_Rα1, and Kv3.2) as well as the spine assessment might support the “cascading” model which claims that cortical areas mature sequentially. In human, spine density increases more rapidly during the second half of gestation and, pruning of synapses during childhood proceeds earlier in auditory cortex than in prefrontal cortex (Huttenlocher and Dabholkar 1997). Thus, the “cascade” starts well before birth and might have been missed by analyses beginning at birth. In line with this, the expression of myelin-related proteins commences 1–2 weeks earlier in prenatal SC than in VC (Sobierajski et al. 2023), suggestive of being driven by specific sensory input, in addition to spontaneous activity of the sensory periphery. Moreover, the myelin sheaths in SC were thicker on average at E100 than those in VC. On the contrary, expression of angiogenesis and tight-junction-related proteins commences at about the same time in SC and VC (Sobierajski et al. 2024) Moreover, several of the synapse-related proteins tested in the present work (e.g. Munc-18, Synapsin1, Trim46, CaMKIIα, and GRIP1) had equal expression levels and developmental profiles in VC and SC. Taken together, neither hypothesis can be fully supported, and it depends on which process and which protein one looks at.

Spontaneous activity and activity evoked by peripheral stimulation is present very early in the ungulate fetal cortex (see discussion in Sobierajski et al. 2024). Expression of synapse-related proteins, production of spines, and spine maturation are regulated by activity. For instance, very early activity in networks extending over millimeters has been reported in ferret cortex (Powell et al. 2024). In sheep, in addition to spontaneous calcium oscillations, optic nerve stimulation at E55 (0.38 gestational age, equals ~ E45 in pig) can elicit visual cortical responses in form of a long-latency surface wave (Persson and Stenberg 1972) which resembles the wave evoked by somatosensory stimulation at E55. The latter wave presumably reflects postsynaptic potentials in basal dendrites of cortical pyramidal neurons of infragranular layers and subplate (Molliver 1967).

The spine assessment yielded the highest numbers in the category of “thin” spines. Numbers might have been a bit overestimated because neck width has been reported to depend on fixation. After cryofixation spine neck width has been found to be 30% thinner (Tamada et al. 2020). Enhancing the difference between neck and head width might affect the classification, however, that would account for all stages equally. Of note, the P5 domestic piglet displayed a total spine density seen at E110 in wild boar, suggesting a delay in spine development. A reason could be that the piglet underwent a 1 h anesthesia to demonstrate a nerve recording to medical students at the University of Heidelberg before euthanasia. In rat prefrontal cortex, perinatal exposure to anesthetics rapidly decreases spine density, whereas exposure around the peak of synaptogenesis rapidly increases spine density without evoking cell death or dendritic reorganization (Briner et al. 2011). Given the advanced development of perinatal wild boar we expected the latter effect. However, the low level in the P5 piglet rather argues for a deficit evoked by domestication.

What innervates the early neurons in SC and VC to promote spinogenesis? First, spines offer docking space for local connections which innervate in particular basal dendrites. Second, specific afferents deliver sensory information to the SC and at least spontaneous activity generated in the retina will arrive in the VC. Spontaneous activity efficiently drives development of the functional architecture (Meister et al. 1991), and its spatial pattern rather than its frequency plays the instructive role in topographic map formation (Xu et al. 2011). Third, immature cortical areas are connected by long-range projections and the activity elicited in one area may easily travel to neighboring areas (Powell et al. 2024). Fourth, transient connections exist e.g. between auditory and visual areas (Clarke and Innocenti 1990), as well as within the areas (Assal and Innocenti 1993). Also, callosal projections exuberantly innervate deeper cortical layers, and even when not maintained into adulthood such connections will exert a modulatory role on functional and morphological maturation (Elberger 1994; Innocenti and Price 2005). The early presence of synaptic vesicle proteins in axons (Watson et al. 2023) and organizers of the pre- and postsynapse as well as receptors for glutamate and GABA suggested a robust increase of synaptic transmission from E65 onwards in pig cortex.

In pig VC and SC, CamKIIα, synaptopodin, and α-actinin-2 were detectable from E65 onwards. Synaptopodin is a marker of spine maturation (Deller et al. 2000) being enriched in mushroom spines. Synaptopodin is phosphorylated by CamKIIα, and can then bind to a 14-3-3 protein which protects synaptopodin from proteolytic degradation (Faul et al. 2008). Synaptopodin mediates long-term spine stability (Okubo-Suzuki et al. 2008; Fester et al. 2009; Yap et al. 2020) by stabilizing actin via interaction with RhoA and cdc42 (Asanuma et al. 2006) and by regulating local calcium signalling (Korkotian et al. 2014). Interaction with α-actinin-2 (Asanuma et al. 2005) is important for dendritic targeting and postsynaptic enrichment of synaptopodin (Kremerskothen et al. 2005).

Midgestation pyramidal neurons in pig cortex barely had basal dendrites and those present were not yet equipped with protrusions. Synaptopodin expression at E65 might serve another structure, the cisternal organelle of the axon initial segment (Peters et al. 1968). Pyramidal cells have axons descending into the white matter from migration onwards. The βIV-spectrin-positive domain characterizing the axon initial segment is well present at E70 in pig cortex pyramidal cells as well as in NPY-positive subplate neurons. Further, the domain elongates substantially until P30 and continuously shifts closer to the soma (Ernst et al. 2018). Studies in dissociated hippocampal neurons have revealed that the axon initial segment acquires a cisternal organelle within a few days after axonal outgrowth, and synaptopodin expression at this early stage is independent of the neuron’s action potential activity (Sánchez-Ponce et al. 2011).

In rodent, CamKIIα expression in rat cortex starts after P7 (Lin and Redmond 2008). Also, synaptopodin increases postnatally reaching adult levels by the second postnatal week (Asanuma et al. 2005; Czarnecki et al. 2005). This increase is presumably driven by neuronal activity (Roth et al. 2001), because synaptopodin expression increases in rodent hippocampus after induction of LTP (Yamazaki et al. 2001). In pig, the activated T286 phospho-CamKIIα was detectable at E100 in SC and weakly in VC which argues for electrically active neurons. Moreover, CamKIIα has a unique function in structurally interacting with GluN2B via α-actinin-2 to regulate LTP (Wang et al. 2011; Curtis et al. 2023). GluN2B and GluN1 were expressed early in fetal pig VC and SC, and GluN2B reached the P90 level at E100 in SC albeit not yet in VC. A functioning spine apparatus is important for LTP and spatial learning (Deller et al. 2003). Single unit recordings in late-fetal postcentral (somatosensory) cortex of the Göttingen minipig have revealed that the action potential activity at E110 is highly similar to spike patterns observed in prepubertal pig (Konda et al. 1979). Response properties of visual cortical cells in newborn sheep are indistinguishable from those in the adult (Clarke et al. 1979). Together, this suggested the presence of LTP-like mechanisms in E100/E110 pig primary sensory cortices.

The inhibitory system also developed from midgestation onwards. GAD expression was relatively low during the fetal period increasing to P90 levels after E100, presumably due to detection limits in protein lysates. In fact, GAD-positive neurons have been detectable in the marginal zone around E60 (Sobierajski et al., unpublished observation), and from E70 onwards GAD-positive neurons and presynaptic boutons innervating pyramidal cells and glutamatergic axonal loop cells of the subplate are well detectable with immunofluorescence (Ernst et al. 2018). Quite surprisingly, markers for fast-spiking neurons such as Kv3.2 and synaptotagmin-2 (Sommeijer and Levelt 2012) were expressed early which confirms the early maturation of interneurons (Ernst et al. 2018). In line, the calcium-binding protein parvalbumin, a marker for basket and chandelier cells, is well expressed at E100, and even more, parvalbumin-positive axons have already been myelinated at this stage (Sobierajski et al. 2023). Similarly, parvalbumin-positive neurons are well developed at birth in guinea pig (Nacher et al. 2000). In human VC, parvalbumin-positive cells develop between gestational weeks 26 to 34 (Cao et al. 1996; Honig et al. 1996). In contrast in rat cortex, parvalbumin comes up during the second postnatal week (Patz et al. 2004) and axons become myelinated earliest during the third week (Micheva et al. 2021).

GluN2B-containing NMDA receptors are important for basal dendritic development (Balu and Coyle 2012; Gonda et al. 2020), and these dendrites grow heavily during late gestation pig cortex. Moreover, in SC at E110, the fraction of mushroom spines which possibly had been stabilized by synaptopodin was at the P90 level. The fraction of mushroom spines may be slightly over- or underestimated due to the limits of spatial resolution. Electron microscopy may add more precision to spine type classification in future studies. Total spine densities in E110 pig resemble numbers reported for P15 rat cortical pyramidal cells (Miller 1988). Comparable results have been reported in guinea pig cortex where dendrites and spine densities are at near-adult dimensions well before birth (Schüz 1981). Behaviorally, this makes sense for an ungulate which needs to orientate and run with the group minutes-to-hours after birth.

## Electronic supplementary material

Below is the link to the electronic supplementary material.

Supplementary Material 1

Supplementary Material 2

## Data Availability

No datasets were generated or analysed during the current study.
