# Supplementary material for: Expression of synaptic proteins and development of dendritic spines in fetal and postnatal neocortex of the pig, the European wild boar Sus scrofa
Source: Brain Struct Funct. 2025 Feb 7;230(2):38. doi: 10.1007/s00429-025-02900-0 (PMC11805786; doi:10.1007/s00429-025-02900-0)
Supplement: Supplementary file 1 — Supplementary Material 1 [file 429_2025_2900_MOESM1_ESM.docx]

**Supplementary Material/ Online Resources**


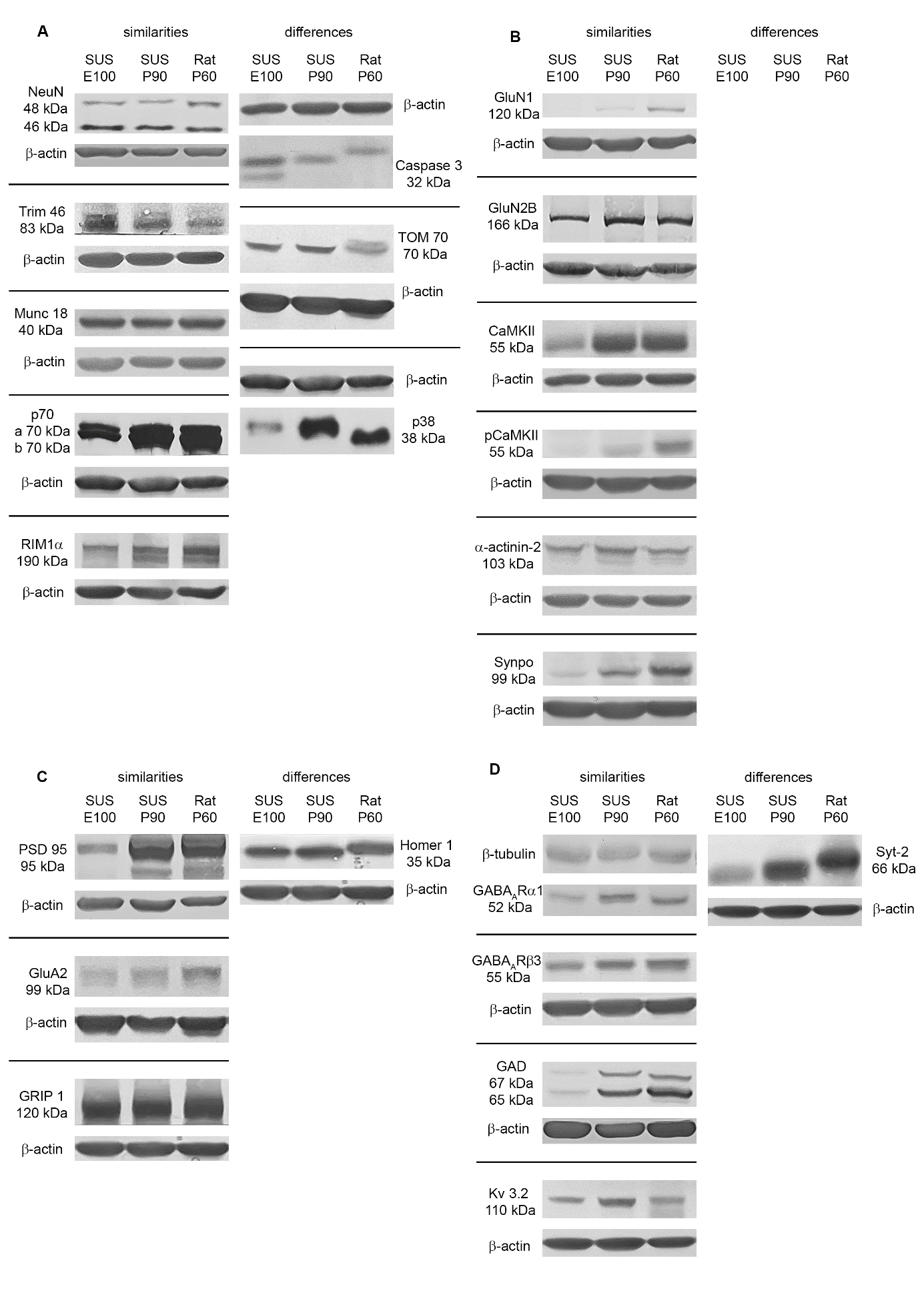
 **Online Resource 1. Comparing protein expression levels of pig to rat VC.** For this arrangement, we selected blots done with VC lysates which differed from those shown in Figures 1-4. We arranged E100, P90 pig and P60 rat to confirm the reliability of the quantitative analysis. The Figure shows the similarities and in some cases differences in band intensity (strength of protein expression) and band size (subtle difference of molecular weight or gel run behaviour). A. Species comparison of proteins shown in Fig.1. B. Species comparison of proteins shown in Fig. 2. C. Species comparison of proteins shown in Fig. 3. D. Species comparison of proteins shown in Fig. 4.

**Online Resource 2**. Antibodies working in rat cortex lysates but failing to yield reliable and measurable bands in pig cortex lysates

| **Primary antibodies** | **Species, label, method** | **Source, order number, RRID** |
| --- | --- | --- |
| Ankyrin G | Guinea pig, WB | Synaptic Systems, Göttingen, DE, Cat# 386 004, RRID: AB_2725774 |
| βIV-Spektrin | Mouse, WB | NeuroMab, Davis, CA, USA, Cat. No. 75-377, AB_2315818 |
| Gephyrin | Mouse, WB | Synaptic Systems, Göttingen, DE, Cat# 147111, AB_887719 |
| GluA1 | Rabbit, WB | Alomone Labs Ltd., Jerusalem, IL Cat# AGC-004, RRID: AB_2039878 |
| GluA2 | Rabbit, WB | Alomone Labs Ltd., Jerusalem, IL Cat# AGC-005, RRID: AB_ 2039881 |
| KIF1α | Rabbit, WB | Abcam, Cambridge, UK, Cat# ab240222, RRID: AB_3107103 |
| Kv3.1b | Rabbit, WB | Synaptic Systems, Göttingen, DE, Cat# 242003, RRID: AB_10804287 |
| GluN2A | Rabbit, WB | Merck Millipore, Burlington, MA, USA, Cat# 07-632, RRID: AB_310837? |
| Tyro-3 | Rabbit, WB | Cell Signaling, Cambridge, UK, Cat# D38C6, RRID: AB_10706782 |
| vGAT | Rabbit, WB | Synaptic Systems, Göttingen, DE, Cat# 131 008, AB_2800534 |
| vGlut | Rabbit, WB | Synaptic Systems, Göttingen, DE, Cat# 135 302, AB_887877 |

Online Resource 3 is online available as separate excel sheet.
